# Genetic relationships between feed efficiency and gut microbiome in pig lines selected for residual feed intake

**DOI:** 10.1111/jbg.12539

**Published:** 2021-02-26

**Authors:** Amir Aliakbari, Olivier Zemb, Yvon Billon, Céline Barilly, Ingrid Ahn, Juliette Riquet, Hélène Gilbert

**Affiliations:** ^1^ GenPhySE Université de Toulouse INRAE Castanet‐Tolosan France; ^2^ GenESI INRAE Surgères France

**Keywords:** feed efficiency, genetic, gut microbiome, heritability, pigs

## Abstract

This study aimed to evaluate the genetic relationship between faecal microbial composition and five feed efficiency (FE) and production traits, residual feed intake (RFI), feed conversion ratio (FCR), daily feed intake (DFI), average daily gain (ADG) and backfat thickness (BFT). A total of 588 samples from two experimental pig lines developed by divergent selection for RFI were sequenced for the 16 rRNA hypervariable V3‐V4 region. The 75 genera with less than 20% zero values (97% of the counts) and two α‐diversity indexes were analysed. Line comparison of the microbiota traits and estimations of heritability (h^2^) and genetic correlations (*r*_g_) were analysed. A non‐metric multidimensional scaling showed line differences between genera. The α‐diversity indexes were higher in the LRFI line than in the HRFI line (*p* < .01), with h^2^ estimates of 0.19 ± 0.08 (Shannon) and 0.12 ± 0.06 (Simpson). Forty‐eight genera had a significant h^2^ (>0.125). The *r*_g_ of the α‐diversities indexes with production traits were negative. Some *r*_g_ of genera belonging to the *Lachnospiraceae*, *Ruminococcaceae*, *Prevotellaceae*, *Lactobacillaceae*, *Streptococcaceae*, *Rikenellaceae* and *Desulfovibrionaceae* families significantly differed from zero (*p* < .05) with FE traits, RFI (3), DFI (7) and BFT (11). These results suggest that a sizable part of the variability of the gut microbial community is under genetic control and has genetic relationships with FE, including diversity indicators. It offers promising perspectives for selection for feed efficiency using gut microbiome composition in pigs.

## 1 INTRODUCTION

Recent advances in bioinformatics and sequencing technologies have made it possible to obtain individual microbiome information for humans, animals and plants. The fundamental role of gut microbiota in essential biological processes such as physiological ageing in humans (Muscogiuri et al., 2019), methane emission in dairy cows, and nutrient digestion, absorption and metabolism of pigs (Niu et al., 2019) makes it a key field of research to counteract major physiological defaults such as obesity in human, and to improve quantitative production traits in livestock. In this regard, measuring the magnitude of genetic control on gut microbiota composition is fundamental to enlighten its potential use in animal selection programs. From a quantitative genetics perspective, estimating heritability (h^2^) quantifies the magnitude of genetic control of a trait. Heritability is a population‐specific parameter that estimates the proportion of additive genetic variance to the phenotypic variance of the trait. Besides the heritability, another essential genetic parameter is the additive genetic correlation (*r*_g_). These two parameters are crucial to predict direct and correlated responses to selection, which are other parameters to evaluate if and how a trait would be affected by selection (Brenner et al., 2002).

In pig breeding, production and feed efficiency (FE) traits, because of their key economic and environmental importance, have a high impact on the sustainability of this industry (Ottosen et al., 2020; Soleimani and Gilbert, 2021). Therefore, research around FE covers a wide range of studies, from traditional statistical methods to recent advances in benefiting from biological data like metabolomics, including few with microbiome information (Maltecca et al., 2020). Several previous studies attempted to discover the link between host genetics, microbiota data, and feed efficiency (Bergamaschi, Maltecca, et al., 2020; Bergamaschi, Tiezzi, et al., 2020; Camarinha‐Silva et al., 2017; McCormack et al., 2017). A study on low and high residual feed intake (RFI) pigs showed a slight difference between the intestinal microbiota of two groups of animals chosen for their phenotypic RFI, and suggested a link between microbial community and FE at the phenotypic level (McCormack et al., 2017). However, direction of correlated responses between RFI and microbiota composition are still unknown. In the present study, we aimed to seek the genetic relationships between five production and FE traits and faecal microbial composition, using data from two experimental pig lines developed by divergent selection for RFI. Statistical analyses were applied to microbiota genera, microbial diversity and performance traits to compare faecal microbiota composition between lines, and h^2^ and *r*_g_ were obtained to describe the transmissible relationships between these traits and microbial traits.

## 2 MATERIALS AND METHODS

### 2.1 Data structure

The data were collected from two experimental French Large White pig lines developed during 10 generations of divergent selection for RFI between 2000 and 2017 at INRAE (UE GenESI, https://doi.org/10.15454/1.5572415481185847E12). The selection process and structure of the data from the two divergent lines have been described in Gilbert et al. (2017) and Aliakbari et al. (2020). Briefly, the G0 individuals were obtained from artificial insemination of 30 sows with 30 boars in generation F0. From the G0 litters, 116 boars were tested for RFI as candidates for selection. Among them 6 extreme low RFI (LRFI) and 6 extreme high RFI (HRFI) boars were selected to be the founders of each line. The selected founder boars were randomly mated with about 70 G0 gilts to initiate the two divergent lines. From generations G1 to G10, the same procedure was implemented within each line, with 96 tested boars per line to produce the next generation. There was no selection on the female side, and sows from both lines were distributed in two farms in equal proportions, which corresponds to two herds of birth for the tested pigs. After weaning, all pigs were gathered on the same farm for testing. In each generation, at least one additional parity was produced to evaluate the correlated responses to selection of growth, feed intake and efficiency and carcass composition traits on females and castrated males (response animals). Candidates to selection were tested from 35 to 95 kg of body weight (BW), whereas for response animals the test ran from 10 weeks of age until slaughter (105 kg BW until G5 and 115 kg BW afterwards). Testing was organized in four pens per contemporary group (CG), and there were at least four CG tested per generation, systematically including both lines. Pigs were penned in groups of 12, per line, and sex when multiple sexes were tested. Pens were equipped with single‐place electronic feeders ACEMA64 (ACEMO, France) to record individual feed intake. A pelleted diet based on cereals and soya bean meal was available ad libitum, and contained 10 MJ net energy (NE)/kg and 160 g CP/kg, with a minimum of 0.80 g digestible Lys/MJ NE. Complete pedigree information was registered, starting at least one generation before F0 ancestors, to G10.

Selection candidates had records for feed intake and feed efficiency traits, growth traits, and live body composition traits. Response animals had records for the same traits recorded from 10 weeks of age until slaughter weight, plus carcass composition traits. In all generations boars were selected based on a phenotypic index combining daily feed intake (DFI) and average daily gain (ADG) between 35 and 95 kg BW, and backfat thickness (BFT) at 95 kg BW (Gilbert et al., 2007), as DFI (g/day)−(1.06 × ADG (g/day))−(37 × BFT (mm)).

For the candidates to selection and the response animals, an RFI was computed as the residual of a multiple linear regression applied to DFI, using realized phenotypic correlations with traits accounting for production requirements (growth rate and body composition) and maintenance requirements (average metabolic BW (AMBW)), and the fixed effects of sex, pen size, CG, and the covariate of BW at the beginning of the test for response animals (Gilbert et al., 2017). Different equations were used for the two groups of animals, to account for the test differences. The RFI equation for selection candidates included ADG and BFT (measured by ultrasounds), and because the test was run between fixed BW, AMBW would be equal for all animals and therefore was skipped from the equation. For response animals, the RFI equation included AMBW, ADG, carcass BFT (carcBFT) and lean meat content (LMC; computed from cut weights). Feed conversion ratio (FCR) was computed based on the corresponding test period of the two groups of animals.

In this study, five phenotypic traits available in both types of animals were studied: RFI, FCR, DFI, ADG, and BFT. To increase the statistical power, given the high *r* *_g_* estimated in preliminary analyses between the traits measured in candidate and response animals, the phenotypic records were combined for both cohorts, after standardization of the records from candidates to selection to the variance of the corresponding trait of the response animals, as described in Aliakbari et al. (2020). Descriptive information of the five traits from G0 to G10 are given in Table 1.

**TABLE 1:** Number (*N*), minimum (Min), maximum (Max), mean and standard deviation (*SD*) of the studied traits in the low residual feed intake (LRFI) and high RFI (HRFI) lines

		*N*	Min	Max	Mean	*SD*	*p*‐value^a^
RFI	LRFI	1,901	−0.38	0.37	−0.04	0.12	^***^
	HRFI	1,748	−0.33	0.39	0.05	0.11	
FCR	LRFI	2,190	1.60	3.88	2.61	0.25	^***^
	HRFI	1,981	2.13	3.93	2.82	0.27	
DFI	LRFI	2,172	1.25	2.92	2.02	0.25	^***^
	HRFI	1,974	1.37	2.97	2.19	0.27	
BFT	LRFI	2,058	9.82	44.63	25.44	7.01	^***^
	HRFI	1,863	9.67	46.76	26.45	7.44	
ADG	LRFI	2,251	0.51	1.02	0.76	0.08	^*^
	HRFI	2,060	0.50	1.01	0.76	0.08	

Abbreviations: ADG, average daily gain (kg/day); BFT, backfat thickness (mm); DFI, daily feed intake (kg/day); FCR, feed conversion ratio (kg/kg); RFI, residual feed intake (kg/day).

^a^ *p*‐value of the effect of the line in a linear model.

^*^ *p*‐value < .05.

^***^ *p*‐value < .001.

### 2.2 Faeces sampling, microbial DNA extraction, 16S rRNA gene sequencing and sequence preprocessing

The microbiota information is most often derived from partial sequencing of the bacterial 16S ribosomal RNA (rRNA) gene, a housekeeping gene in all bacteria (Woese, 1987). Sequencing the 16S rRNA gene has become a standard approach in bacterial taxonomic classification, due to its ease to generate phylogenetic information at high throughput (Wang et al., 2015). For this purpose, nine hypervariable regions (V1‐V9) of the 16S rRNA gene can be targeted for sequencing. Sequences can then be analysed as separate Amplicon Sequence Variant (ASV), or clustered into “Operational Taxonomic Units” (OTUs) based on their similarities. The ASV approach enables easier comparison between studies (Callahan et al., 2017). These units allow inferring the taxonomy of species present in the targeted biological samples using several reference databases. The counts of each OTU or ASV throughout the samples form a matrix called abundance table that is the basis of downstream analyses. Faecal sampling is a convenient and non‐invasive sampling method that provides a reasonably good representation of the gut microbial communities (Ingala et al., 2018). It is now more common than other sampling locations for profiling of microbial communities in large mammalian animal populations.

For our study, faecal samples of 604 animals from G9 and G10 of the LRFI and HRFI lines were collected at 15 weeks of age, homogenized and placed immediately in dry ice, before storage at −80°C. The animals collected in G9 were the boars candidate to selection, and the pigs in G10 were females and castrated males response to selection. Microbial profiling was done as described previously (Achard et al., 2020). Briefly, the microbial DNA was extracted using the Quick‐DNA^™^ Faecal Microbe Miniprep Kit^™^ (Zymo Research) and a 15 min bead‐beating step at 30 Hz was applied. The V3‐V4 region was then amplified from diluted genomic DNA with the primers F343 (CTTTCCCTACACGACGCTCTTCCGATCTTACGGRAGGCAGCAG) and R784 (GGAGTTCAGACGTGTGCTCTTCCGATCTTACCAGGGTATCTAATCCT) using 30 amplification cycles with an annealing temperature of 65°C. This V3‐V4 region has proved useful to study the variability of the pig microbiota in previous studies (Le Floc'h et al., 2014; Verschuren et al., 2018). The ends of each read overlap and can be stitched. In a single run, it generates extremely high quality, full‐length reads of the full V3 and V4 region. The Flash software v1.2.6 (Magoc & Salzberg, 2011) was used to assemble each pair‐end sequence, with at least a 10‐bp overlap between the forward and reverse sequences, allowing 10% mismatch. Single multiplexing was performed using an in‐house 6 bp index*,* which was added to R784 during a second PCR with 12 cycles using forward primer (AATGATACGGCGACCACCGAGATCTACACTCTTTCCCTACACGAC) and reverse primer (CAAGCAGAAGACGGCATACGAGAT‐index‐GTGACTGGAGTTCAGACGTGT). The resulting PCR products were purified and loaded to the Illumina MiSeq cartridge following the manufacturer's instructions. Run quality was internally checked using PhiX, and each pair‐end sequence was assigned to its sample using the integrated index, with the bcl2fastq Illumina software. The sequences were submitted to the Short‐Read Archive with accession number PRJNA701065. Filtering and trimming of sequences of high quality was applied to the reads with the DADA2 package in the R software (Callahan et al., 2016) with the following parameters: maxN = 0, maxEE = 2, truncQ = 2, trimleft = 17. Chimera were removed with the consensus method to obtain the final OTU abundance table. No further clustering was applied, so OTU were equivalent to ASV in this study. This step was followed by taxonomic annotation using the assignTaxonomy function of dada2 with the Silva Dataset v132 (Quast et al., 2013).

The final abundance table was rarefied to 9,000 counts per sample, and contained 5,689 OTUs or 298 genera across 604 samples. The 16 samples that contained fewer reads than 9,000 were discarded, resulting in 588 samples in the final abundance table, 295 LRFI and 293 HRFI pigs. The microbiota analyses were then run at the genus level. The OTU relative abundances with the same taxonomic path until an identical genus were thus aggregated in a single count. Counts belonging to unclassified genera of a family were systematically gathered into a pseudo genus named NA_Family.

In addition, to limit the deviations of the genera distribution from the Gaussian distribution assumption used in linear mixed models (see next section), the genera table was filtered for a maximum proportion of 20% zero abundancy for each genus, and the resulting abundancies were log‐transformed after adding a constant value of 1 to all counts. After this filtration step, 75 genera remained for the downstream analyses. Finally, to better understand how the genera are distributed, two α‐diversity metrics, the Shannon (Shannon, 1948) and Simpson (Simpson, 1949) metrics, were calculated from the filtered table with 75 genera, and analysed as additional individual microbial traits.

### 2.3 Statistical analyses

The beta‐diversity is usually used to demonstrate the community differentiation between cohorts (Whittaker, 1960). To represent the beta‐diversity between the faecal microbial genera communities of both lines, a non‐metric multidimensional scaling (NMDS) based on the Bray–Curtis dissimilarity distance matrix was applied to the abundance table. This analysis was done using the R software and package “vegan” (Oksanen et al., 2013). The individual loadings were retrieved for each sample for the two first dimensions of the NMDS. Then, the line effect was tested with a generalized linear model (GLM) on the loadings of the first two dimensions of the NMDS, the α‐diversity metrics, the genera abundances, and the production traits. In addition, contributions of the genera to each axis, and to the plan defined by the two first axes, were computed as the squares of the loadings and sum of squares of the loadings, respectively. Before testing the line differences, variables with positive values (counts and diversity indexes) were log‐transformed, whereas the loadings of the NMDS that contained negative values were submitted to a Johnson transformation (Johnson, 1949). These analyses were performed using package “car” in the R software (Fox et al., 2012) and the line effect was declared significant for *p* < .05 for the corresponding *F*‐test.

Following the main objective of the study, searching for the genetic relationships between microbiota traits and FE traits required the estimations of (co)variance components. The best linear unbiased prediction (BLUP) method was applied to the filtered genera and the two α‐diversity metrics to obtain the (co)variance components. To follow the assumption of the BLUP method, which should be applied to a non‐selected base population, all analyses were done in bivariate models including the selection index as the first trait. The second trait was the microbiota observation vector (abundance of each genus or α‐diversity metric). To compute genetic correlations between the performance traits and microbiota observations, each of the production traits was added in three‐variate analyses.

The significance of fixed environmental factors (*p* < .05) on all response variables was tested in preliminary GLM analyses. Significant fixed factors, including pen size (5 levels), herd of birth (two levels), sex (three levels), and contemporary group (CG, 109 levels) for performance traits, microbiota data and α‐diversity metrics, were systematically fitted. The fitted covariates were slaughter body weight (BW) for BFT and BW at test for genera abundancies and α‐diversity metrics. The significance of all fitted fixed factors on the 75 genera are given in Table S1. The litter effect was fitted as a random environmental source of variation for performance traits, and for microbiota data whenever it was significant (*p* < .05 for a χ^2^ test applied to the likelihood ratio test comparing the models with and without this term).

The following bivariate and three‐variate animal models were used to estimate the variance components:

$$y=\mathrm{Xb}+Z_{1}a+Z_{2}l+e$$

where ***y*** is the vector of observations for the index and the abundance of each genus or an α‐diversity metric, and one of five performance traits (in three‐variate analyses), ***b*** is the vector of fixed effects (described above), ***a*** is the vector of additive genetic effects, ***l*** is the vector of litter effects, and ***e*** is the vector of random residuals. ***X***, ***Z*_1_** and ***Z*_2_** are the incidence matrices for ***b***, ***a*** and ***l***. The distributions assumed for the random terms were ***a*** ~ *N* (0, **G_0_** ⊗ **A**), ***l*** ~ *N* (0, **R_l_** ⊗ **I**), and ***e*** ~ *N* (*0,* **R_e_** ⊗ **I**), where **G_0_** is a 2 × 2 or 3 × 3 symmetrical direct additive genetic effect (co)variance matrix, and **R_l_** and **R_e_** are 2 × 2 or 3 × 3 symmetrical litter effect and residual effect (co)variance matrices, respectively. **I** denoted the identity matrix of adequate dimension. The pedigree relationship matrix (**A**) included 10 generations of pedigree information plus ancestors, and contained 7,293 animals. The analyses were performed using AIREMLF90 software (Misztal et al., 2018) for the BLUP method.

To test the significance of h^2^ of the 75 genera, an empirical significance threshold equal to 0.125 was considered. The threshold was obtained after running 10,000 univariate analyses using the above described genetic model applied to microbiota abundancies, based on a null hypothesis of no genetic control on the abundancies. The null hypothesis was obtained by shuffling the abundances across individuals for two arbitrary genera. The minimum value of the top 5% of the estimated h^2^ was considered as the threshold to decide that a genus was heritable. Thereafter, the three‐variate analyses were conducted for genera with h^2^ significantly different from zero. The deviation from zero of the additive *r*_g_ of genera and α‐diversity metrics with the production traits were tested using a *Z*‐test.

## 3 RESULTS

### 3.1 Gut microbiome differences between lines

**FIGURE 1:**
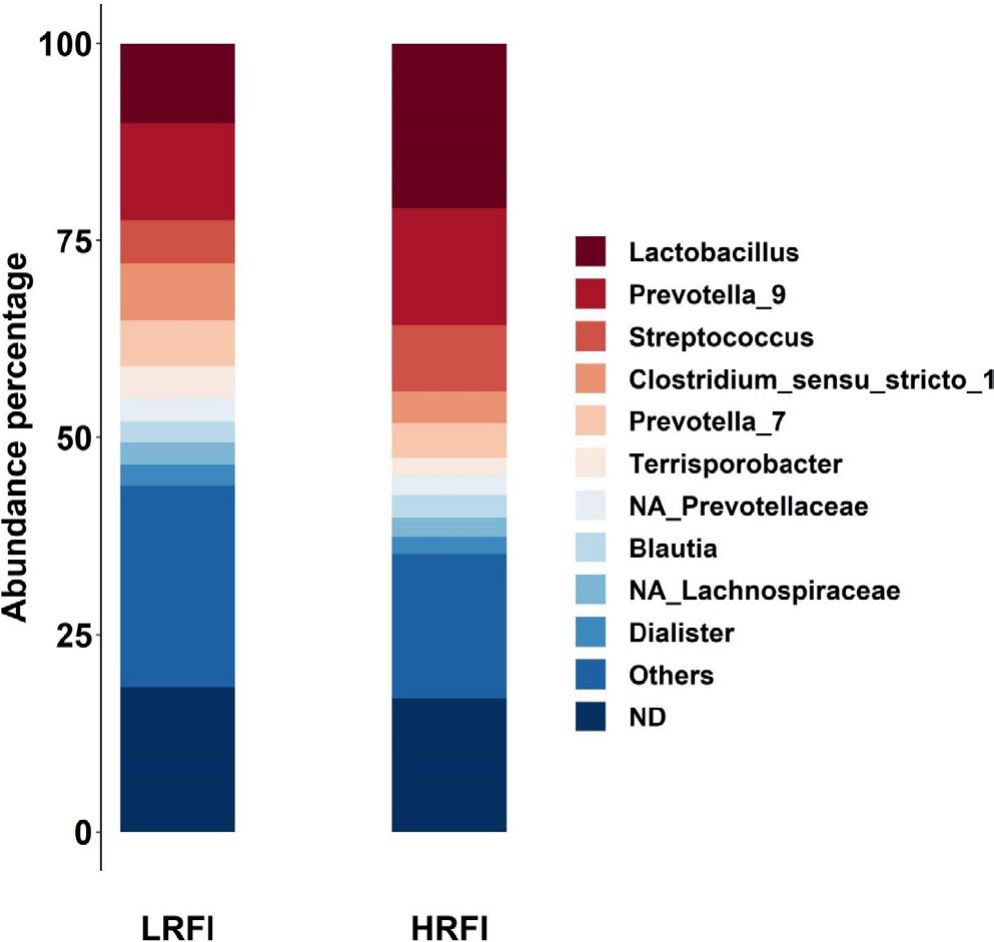
Abundance percentage of the 75 genera in the LRFI and HRFI lines. ***Others =*** differentially abundant genera between lines with abundances lower than 2%, ***ND*** = genera with non‐significant abundance difference between the two lines [Colour figure can be viewed at wileyonlinelibrary.com]

The 75 filtered genera represented on average 97% of the sample counts of the rarefied table. Among these genera, 42 had significantly higher abundances in the LRFI line than in the HRFI line, and 10 were more abundant in the HRFI line (Figure 1 and Table S2). Of the differentially abundant genera between lines (*p* < .05 for a Student test applied to the log‐transformed abundances), the genera *Lactobacillus* (10.1% in the LRFI line and 20.9% in the HRFI line of the 75 genera counts, *p* < .0001), *Prevotella_9* (12.2% and 14.8% in the LRFI and HRFI lines, respectively, *p* < .03), and *Streptococcus* (5.6% in the LRFI line versus 8.5% in the HRFI line, *p* < .0001) were the more abundant genera in both lines, and they were all more abundant in the HRFI line. The three genera *Clostridium_sensu_stricto_1* (*p *< .0001), *Prevotella_7* (*p* < .004), and *Terrisporobacter* (*p* < .0001) were more abundant in the LRFI line (7.2%, 5.7% and 4.1%, respectively) than in the HRFI line (4.0%, 4.4% and 2.3%, respectively). The four genera *Dialister* (*p* < .05), *NA_Prevotellaceae* (*p* < .0001), *NA_Lachnospiraceae* (more abundant in the LRFI line, *p* < .0001), and *Blautia* (more abundant in the HRFI line, *p* < .0001) represented on average 2.2% of the counts. The other 42 differentially abundant genera had abundances lower than 2% in the two lines, and represented a total of 25.9% and 18.6% of the abundances in the LRFI and HRFI lines, respectively. The remaining 23 genera that were not significantly different (*p* > .05) between the lines had total abundance of 16.2% in the LRFI and 14.6% in the HRFI lines.

**FIGURE 2:**
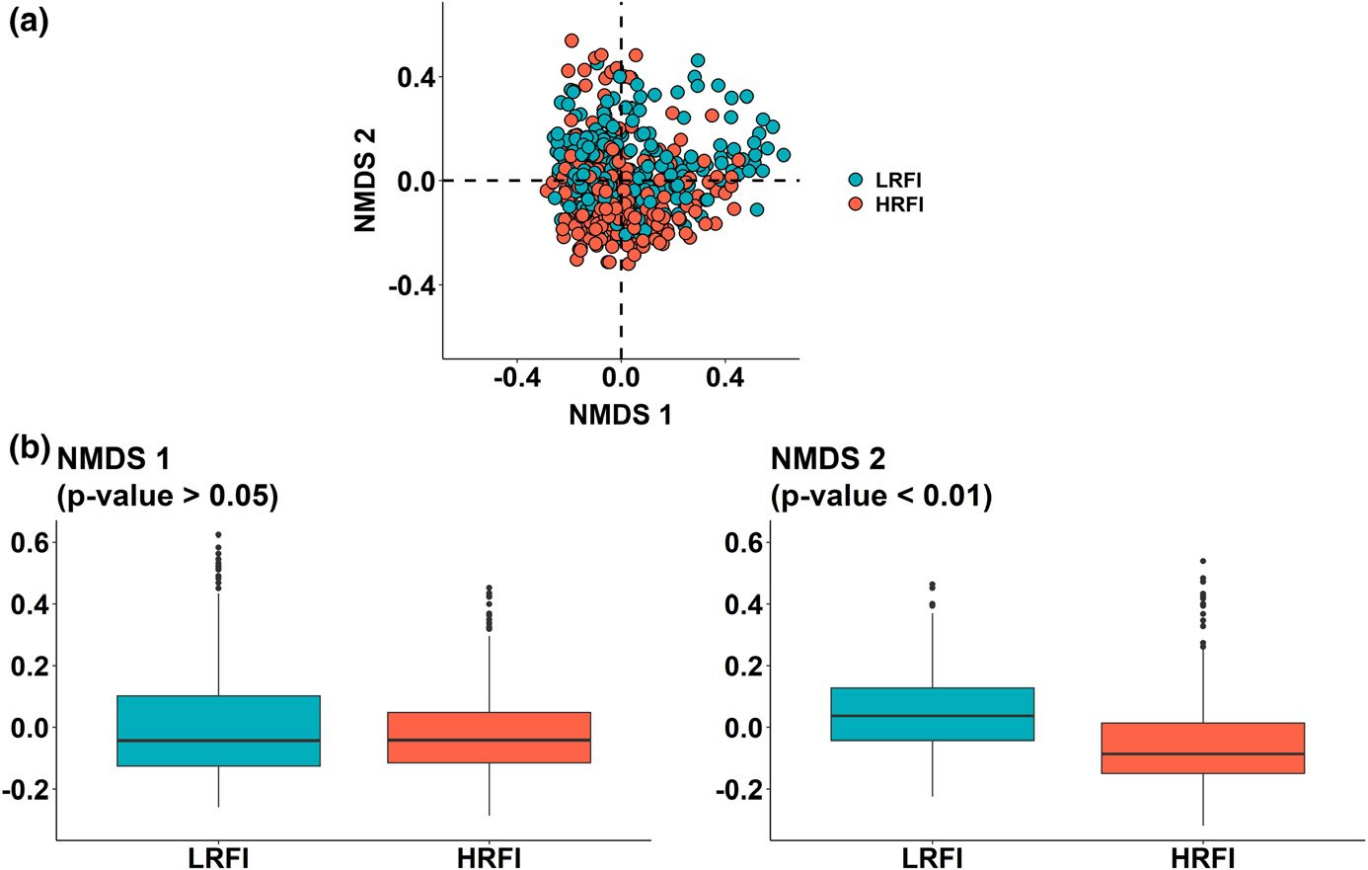
Non‐metric multidimensional scaling (NMDS) based on the Bray–Curtis dissimilarity matrix of the genera community (a) and box plots of the individual coordinates per line on the two first axes of the NMDS (LRFI, low residual feed intake; HRFI, high residual feed intake), with *p*‐value of the ANOVA test of the line differences (b) [Colour figure can be viewed at wileyonlinelibrary.com]

**FIGURE 3:**
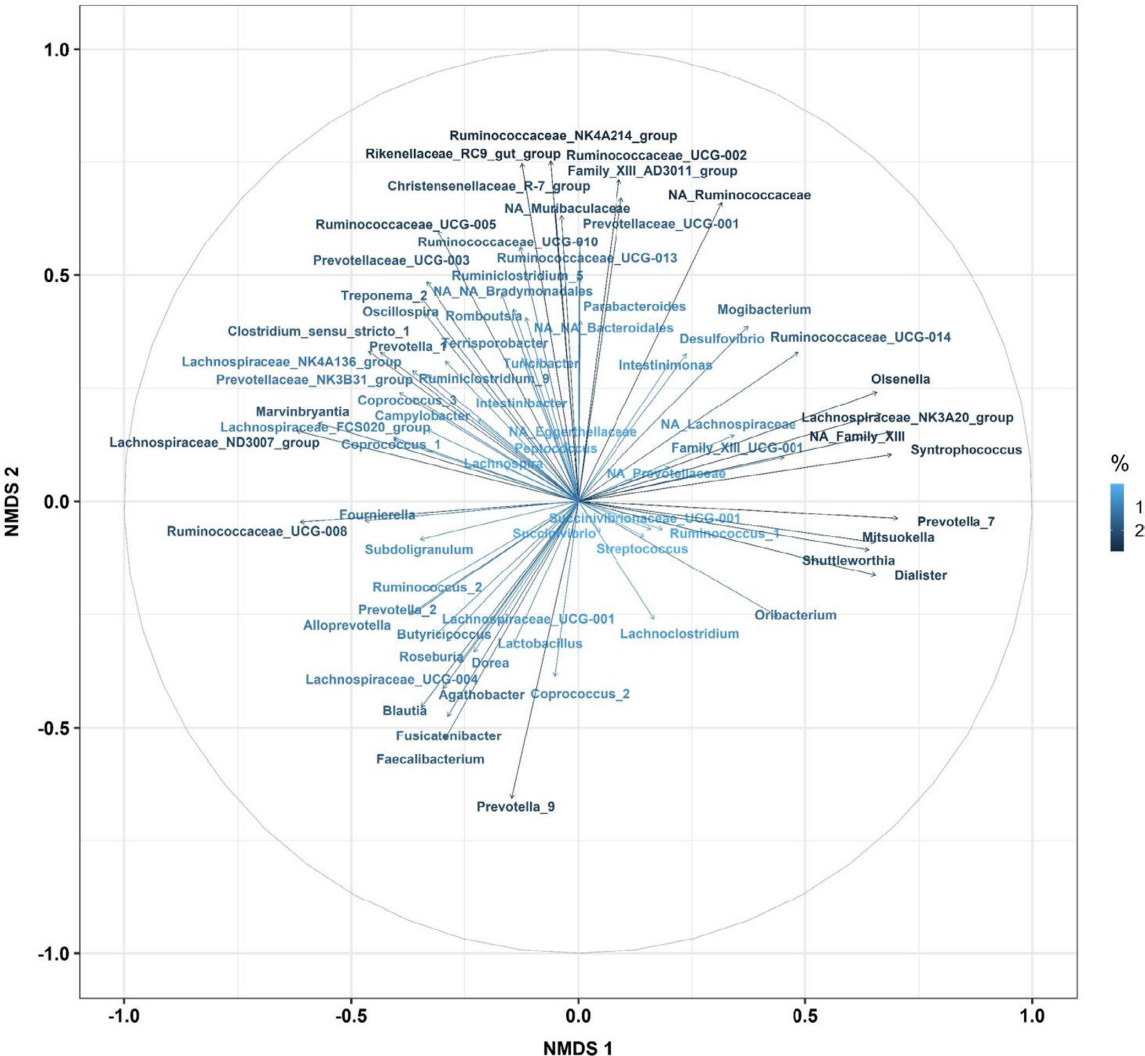
Projection of the genera on the first and second dimensions in a non‐metric multidimensional scaling (NMDS) applied to the Bray–Curtis matrix of the genera abundances. The arrows are coloured based on the contribution of each genus to the plan [Colour figure can be viewed at wileyonlinelibrary.com]

The NMDS showed differences between the genera communities of the LRFI and HRFI lines (Figure 2). The two lines were significantly differentially distributed only along the second (*p* < .01) dimension. Among the 75 genera included in the NMDS, the genus *Rikenellaceae_RC9_gut_group* had the highest (2.9%) contribution to the plan defined by dimensions 1 and 2, and the genus *Succinivibrio* had the lowest (0.03%) contribution (Table S2 and Figure 3). In details, on the first axis 25 genera had a contribution larger than the expected contribution if all genera contributed equally 1.33% (100/75), including 15 genera differentially abundant between the lines. It was mainly driven (contributions larger than 3.2%) by the opposition of the genera *Prevotella_7* (5.2%), *Syntrophococcus* (5.0%), *NA_Family_XIII* (5.0%), *Lachnospiraceae_NK3A20_group* (4.7%), *Olsenella* (4.6%), *Dialister* (4.5%), *Mitsuokella* (4.5%), and *Shuttleworthia* (4.3%) in one direction, and the genera *Lachnospiraceae_ND3007_group* (4.0%), *Ruminococcaceae_UCG‐008* (3.9%), and *Marvinbryantia* (3.4%) in the other direction. On the second axis, 25 genera had contributions larger than 1.33%, including 22 genera differentially abundant between the lines. The genera *Prevotella_9* (4.3%) drove the direction towards more HRFI samples, whereas the genera *Ruminococcaceae_NK4A214_group* (5.7%), *Rikenellaceae_RC9_gut_group* (5.6%), *Ruminococcaceae_UCG‐002* (5.1%), *Family_XIII_AD3011_group* (4.5%), *NA_Ruminococcaceae* (4.4%), *Christensenellaceae_R‐7_group* (4.2%), *NA_Muribaculaceae* (4.0%), *Ruminococcaceae_UCG‐005* (3.6%), *Prevotellaceae_UCG‐001* (3.3%) and *Ruminococcaceae_UCG‐010* (3.2%) were the main contributors to the opposite direction, towards the LRFI line (contributions higher than 3.2%).

**FIGURE 4:**
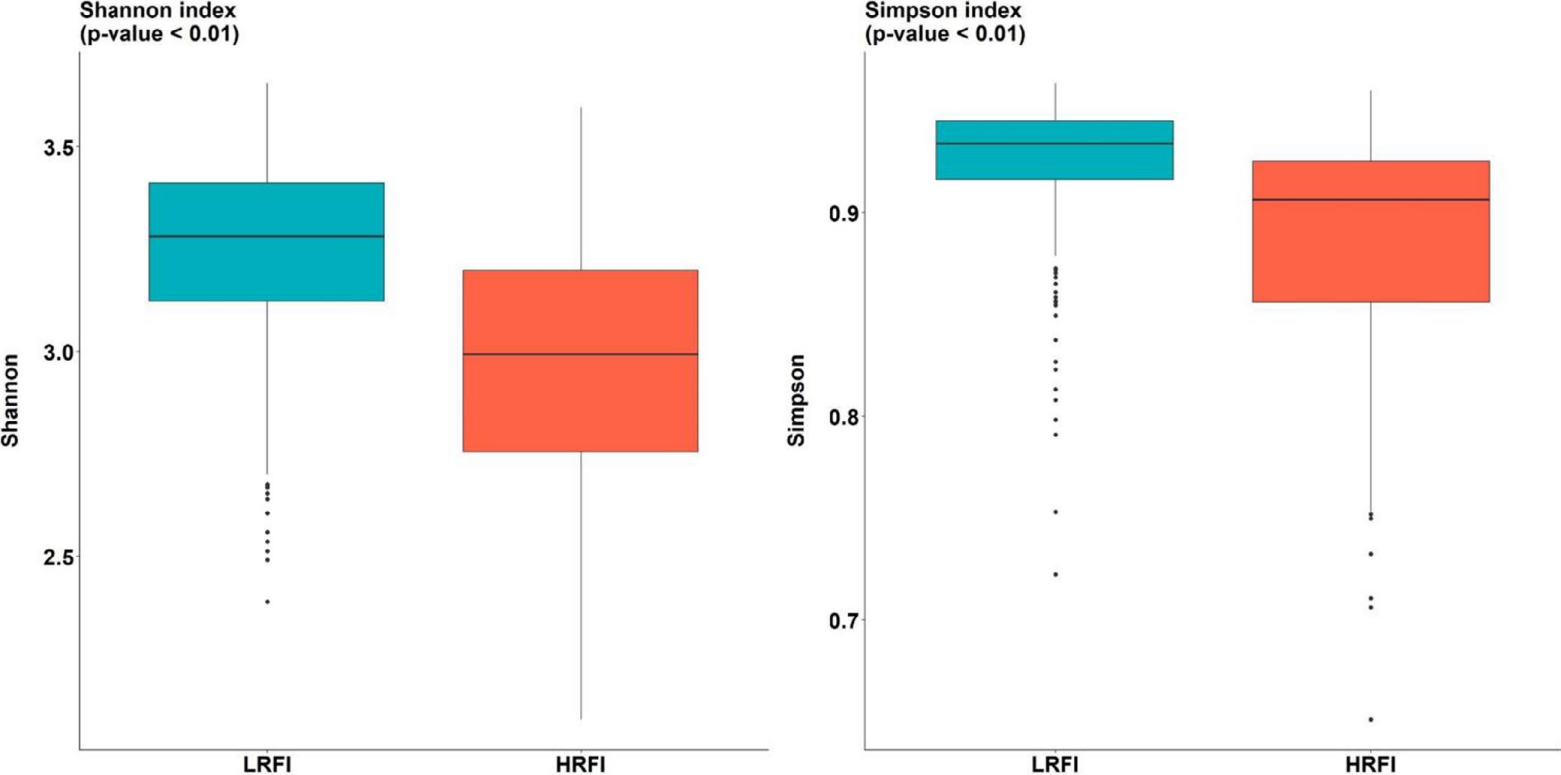
Box plots of Shannon and Simpson α‐diversity indexes per line (LRFI, low residual feed intake (*n* = 295); HRFI, high residual feed intake (*n* = 293)) and *p*‐value of ANOVA test of the line differences [Colour figure can be viewed at wileyonlinelibrary.com]

The Shannon and Simpson α‐diversities indexes showed significantly higher microbial diversity in the LRFI line than in the HRFI line (*p* < .01, Figure 4).

### 3.2 Heritability estimates of microbiota traits

**FIGURE 5:**
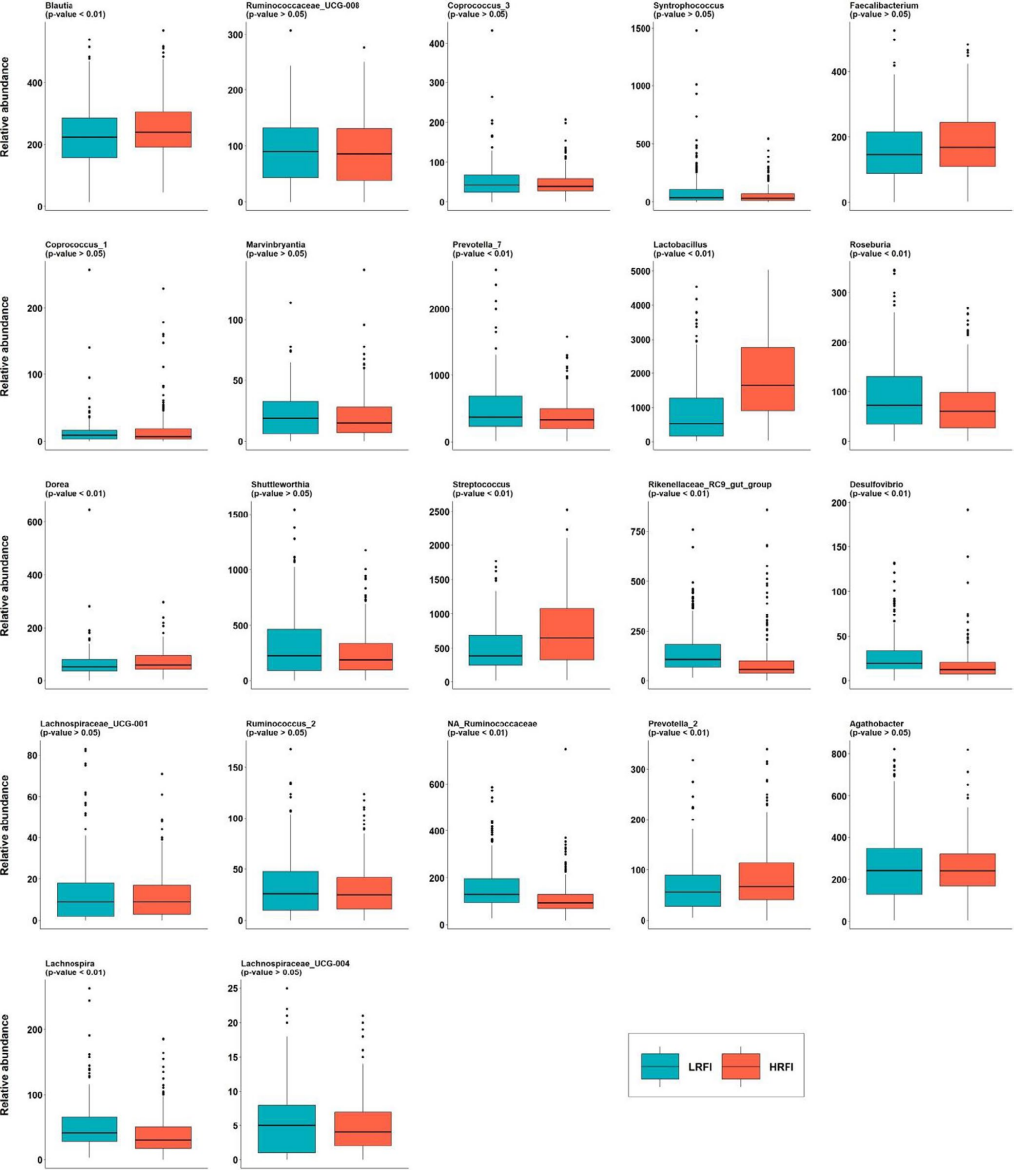
Box plots of genera abundances per line (LRFI, low residual feed intake; HRFI, high residual feed intake) and *p*‐value of ANOVA test of the line differences

The gut microbiota composition can be highly heritable in pigs, but not for all genera. The h^2^ estimates for the Shannon and Simpson α‐diversity indexes were 0.19 ± 0.08 and 0.12 ± 0.06, respectively (Table 2). The estimated h^2^ of the genera ranged from null to 0.50 ± 0.12 for *Clostridium_sensu_stricto_1*. Forty‐eight genera had a h^2^ higher than 0.125, and therefore were considered as heritable, including 34 genera with h^2^ larger than 0.20. The majority of the genera that were differentially abundant between lines were heritable (33/52). Out of the 23 genera that did not differ between lines, 15 had significant h^2^. For the 48 heritable genera, the abundances per line are shown in Table S2 and Figure 5.

**TABLE 2:** Estimated heritability (h^2^) and standard errors (SE) and descriptive statistics (minimum (Min), maximum (Max), mean and standard deviation (*SD*)) of α‐diversity indexes and genera abundances

	h^2^ ± SE^a^	% Zeros	Min	Max	Mean	*SD*
α‐diversity index						
Shannon	0.19 ± 0.08	0	2.1	3.6	3.1	0.3
Simpson	0.12 ± 0.06	0	0.6	1.0	0.9	0.05
Genus						
*Clostridium_sensu_stricto_1*	0.50 ± 0.12	0	2	2,574	488.6	442.9
*Prevotella_1*	0.44 ± 0.11	14	0	306	35.3	45.5
*Blautia*	0.39 ± 0.11	0	14	571	241.5	99.9
*Prevotellaceae_NK3B31_group*	0.36 ± 0.10	2	0	1,216	84.6	131.1
*Lachnospiraceae_NK3A20_group*	0.36 ± 0.10	2	0	1,663	77.7	186.8
*Ruminococcaceae_UCG‐008*	0.35 ± 0.11	2	0	307	89.1	58.7
*Lachnospiraceae_ND3007_group*	0.35 ± 0.11	3	0	109	24.4	17.0
*Coprococcus_3*	0.35 ± 0.10	1	0	432	48.4	37.1
*Butyricicoccus*	0.34 ± 0.11	4	0	68	18.2	12.4
*Terrisporobacter*	0.34 ± 0.11	0	13	1,070	279.0	193.7
*Syntrophococcus*	0.34 ± 0.10	6	0	1,480	74.4	119.9
*Faecalibacterium*	0.33 ± 0.11	0	1	527	169.1	98.3
*Coprococcus_1*	0.32 ± 0.10	13	0	257	14.7	24.4
*Marvinbryantia*	0.30 ± 0.10	5	0	141	20.7	17.9
*Mitsuokella*	0.30 ± 0.09	1	0	1,083	93.6	110.7
*NA_Family_XIII*	0.29 ± 0.09	5	0	285	24.1	36.2
*Prevotella_7*	0.28 ± 0.10	0	12	2,583	441.7	335.5
*Prevotellaceae_UCG‐003*	0.28 ± 0.10	3	0	240	18.9	24.4
*Romboutsia*	0.28 ± 0.10	4	0	235	36.0	39.9
*Fusicatenibacter*	0.27 ± 0.10	3	0	117	24.8	16.8
*Campylobacter*	0.27 ± 0.10	4	0	266	23.6	26.9
*Olsenella*	0.27 ± 0.09	8	0	735	42.6	87.5
*Oscillospira*	0.25 ± 0.09	14	0	85	9.7	10.6
*Lactobacillus*	0.24 ± 0.09	0	17	5,034	1,353.5	1,148.3
*Roseburia*	0.23 ± 0.10	2	0	346	79.5	64.2
*Succinivibrionaceae_UCG‐001*	0.23 ± 0.09	13	0	1,153	94.5	161.2
*NA_Muribaculaceae*	0.23 ± 0.08	0	0	727	68.0	73.1
*Dorea*	0.22 ± 0.09	1	0	648	67.8	47.8
*Subdoligranulum*	0.22 ± 0.09	0	11	583	175.2	93.2
*Alloprevotella*	0.22 ± 0.09	0	3	389	86.1	54.8
*Ruminococcaceae_UCG‐014*	0.22 ± 0.09	0	3	505	108.9	71.9
*Dialister*	0.20 ± 0.08	0	3	844	213.0	135.5
*Shuttleworthia*	0.20 ± 0.09	0	1	1,543	280.7	250.3
*Streptococcus*	0.20 ± 0.10	0	20	2,526	613.1	446.4
*NA_Prevotellaceae*	0.20 ± 0.09	0	2	817	233.6	102.9
*Rikenellaceae_RC9_gut_group*	0.20 ± 0.09	0	0	862	118.3	116.0
*Lachnospiraceae_NK4A136_group*	0.19 ± 0.08	5	0	253	21.6	23.0
*Desulfovibrio*	0.18 ± 0.09	1	0	192	21.5	20.8
*Lachnospiraceae_UCG‐001*	0.18 ± 0.08	14	0	83	12.0	12.7
*Ruminococcus_2*	0.17 ± 0.09	6	0	168	31.1	26.8
*NA_Ruminococcaceae*	0.16 ± 0.08	0	19	749	135.5	89.3
*Treponema_2*	0.16 ± 0.08	7	0	761	58.4	98.2
*Fournierella*	0.14 ± 0.08	13	0	66	9.0	9.1
*Prevotella_2*	0.14 ± 0.08	0	0	340	75.0	56.0
*Agathobacter*	0.14 ± 0.07	0	5	823	253.7	148.1
*Lachnospira*	0.13 ± 0.07	1	0	263	44.0	33.0
*Ruminococcaceae_UCG‐005*	0.13 ± 0.07	0	0	665	72.6	73.2
*Lachnospiraceae_UCG‐004*	0.13 ± 0.07	16	0	25	5.3	4.6
*Ruminococcaceae_UCG‐013*	0.12 ± 0.07	14	0	73	7.8	8.6
*Intestinimonas*	0.10 ± 0.06	5	0	38	7.5	5.5
*Turicibacter*	0.10 ± 0.03	6	0	246	31.7	37.2
*Intestinibacter*	0.09 ± 0.08	0	1	258	39.5	24.3
*Oribacterium*	0.09 ± 0.06	1	0	151	42.9	24.5
*Ruminiclostridium_5*	0.08 ± 0.07	6	0	47	8.1	6.4
*Family_XIII_AD3011_group*	0.08 ± 0.06	1	0	303	37.0	33.7
*Christensenellaceae_R‐7_group*	0.07 ± 0.06	1	0	933	52.3	99.8
*Lachnospiraceae_FCS020_group*	0.07 ± 0.06	2	0	43	11.1	6.8
*NA_NA_Bradymonadales*	0.06 ± 0.06	19	0	356	23.1	37.6
*Family_XIII_UCG‐001*	0.06 ± 0.06	2	0	45	15.2	8.9
*Mogibacterium*	0.06 ± NE	5	0	130	12.9	12.6
*Succinivibrio*	0.05 ± 0.05	5	0	501	29.6	46.1
*NA_Eggerthellaceae*	0.05 ± 0.06	10	0	30	6.2	5.0
*Ruminiclostridium_9*	0.04 ± 0.05	7	0	36	7.3	5.8
*Lachnoclostridium*	0.04 ± 0.05	7	0	140	11.8	11.7
*Ruminococcaceae_NK4A214_group*	0.03 ± 0.01	1	0	244	36.4	35.2
*Ruminococcaceae_UCG‐002*	0.02 ± 0.01	0	1	584	69.4	67.5
*NA_Lachnospiraceae*	0.02 ± 0.01	0	62	661	226.0	76.3
*Ruminococcaceae_UCG‐010*	0.02 ± 0.01	2	0	970	40.9	76.1
*Prevotellaceae_UCG‐001*	0.01 ± NE	19	0	128	8.0	14.5
*Prevotella_9*	0.01 ± NE	0	34	2,935	1,180.6	561.1
*NA_NA_Bacteroidales*	0.01 ± NE	5	0	204	15.9	24.1
*Coprococcus_2*	0.00 ± NE	7	0	81	15.4	12.9
*Peptococcus*	0.00 ± NE	2	0	62	14.0	8.0
*Ruminococcus_1*	0.00 ± NE	0	27	408	143.1	51.9
*Parabacteroides*	0.00 ± NE	12	0	247	12.8	21.6

Abbreviation: NE, not estimable.

^a^ h^2^ were obtained after log transformation.

Heritable genera were also more abundant genera, while non‐heritable genera tended to be at lower abundance (*p* < .05 for a Student test applied to the average of log‐transformed abundances). A Spearman correlation of 0.26 (*p* < .05) was estimated between the h^2^ estimates and the average of log‐transformed abundances, while a correlation of 0.10 (*p* > .05) was obtained with the raw averages.

Comparison of the contributions of the heritable and non‐heritable genera to the axes of NMDS showed a significant difference (*p* < .05) of contribution to the first axis between the two groups of genera: the average contribution of the heritable genera to axis 1 was 1.8%, whereas the non‐heritable genera had an average contribution of 0.5%. The two groups of genera similarly contributed to the second axis (*p* = .08): the average contribution of the heritable and non‐heritable genera to the second axis were 1.1% and 1.8%, respectively.

### 3.3 Genetic correlations of microbiota traits with production traits

The two α‐diversities indexes and 48 genera with significant h^2^ were included in three‐variate analyses to estimate genetic correlations with production traits. The *r*_g_ of the α‐diversities indexes with production traits were negative and similar for the two metrics (Table  3). With ADG, DFI, and RFI, *r*_g_ estimates were lower than 0.27, and did not differ from zero. The highest *r*_g_ were obtained with BFT (*r*_g_ < −0.89 ± 0.04) and FCR (*r*_g_ < −0.61 ± 0.52).

**TABLE 3:** Genetic correlations^a^ (SE) of α‐diversity indexes and genera with production traits

	RFI	FCR	DFI	BFT	ADG
α‐diversity index					
Shannon	−0.26 ± 0.29	−0.61 ± 0.52	−0.30 ± 0.29	−0.89 ± 0.04^a^	−0.21 ± 0.32
Simpson	−0.27 ± 0.34	−0.93 ± NE	−0.42 ± 0.34	−0.94 ± NE	−0.31 ± 0.48
Genus					
*Blautia*	0.20 ± 0.12	0.32 ± 0.23	0.33 ± 0.25	0.50 ± 0.22^a^	0.02 ± 0.26
*Ruminococcaceae_UCG‐008*	0.05 ± 0.23	0.26 ± 0.23	0.32 ± 0.23	0.54 ± 0.22^a^	−0.01 ± 0.28
*Coprococcus_3*	−0.03 ± 0.24	0.27 ± 0.22	0.25 ± 0.21	0.56 ± 0.21^a^	−0.12 ± 0.27
*Syntrophococcus*	−0.04 ± 0.25	−0.18 ± 0.26	−0.29 ± 0.23	−0.60 ± 0.23^a^	−0.03 ± 0.28
*Faecalibacterium*	0.20 ± 0.12	0.26 ± 0.30	0.60 ± 0.12^a^	0.41 ± 0.33	0.18 ± 0.32
*Coprococcus_1*	−0.09 ± 0.25	0.18 ± 0.25	0.30 ± 0.23	0.54 ± 0.24^a^	0.12 ± 0.29
*Marvinbryantia*	0.10 ± 0.24	0.19 ± 0.25	0.29 ± 0.28	0.47 ± 0.24^a^	−0.04 ± 0.29
*Prevotella_7*	−0.19 ± 0.13	−0.11 ± 0.27	−0.28 ± 0.32	−0.71 ± 0.28^a^	−0.08 ± 0.31
*Lactobacillus*	0.29 ± 0.24	−0.05 ± 0.19	0.51 ± 0.34	0.86 ± 0.05 ^a^	0.30 ± 0.35
*Roseburia*	0.01 ± 0.14	−0.05 ± 0.32	0.35 ± 0.12^a^	0.16 ± 0.50	0.31 ± 0.65
*Dorea*	0.14 ± 0.16	0.05 ± 0.47	0.33 ± 0.43	0.66 ± 0.29^a^	0.10 ± 0.40
*Shuttleworthia*	−0.13 ± 0.14	−0.05 ± 0.34	−0.51 ± 0.10^a^	−0.76 ± 0.36^a^	−0.28 ± 0.40
*Streptococcus*	0.32 ± 0.13^a^	−0.24 ± 0.31	−0.17 ± 0.13	−0.49 ± 0.39	−0.38 ± 0.57
*Rikenellaceae_RC9_gut_group*	−0.14 ± 0.29	−0.12 ± 0.37	−0.43 ± 0.38	−0.86 ± 0.06^a^	−0.45 ± 0.44
*Desulfovibrio*	−0.30 ± 0.13^a^	−0.35 ± 0.65	−0.63 ± 0.45	−0.97 ± 0.01^NE^	−0.30 ± 0.52
*Lachnospiraceae_UCG‐001*	−0.01 ± 0.33	−0.03 ± 0.36	0.55 ± 0.12^a^	0.39 ± 0.42	0.73 ± 0.76
*Ruminococcus_2*	0.08 ± 0.14	−0.14 ± 0.48	0.44 ± 0.12^a^	0.14 ± 0.49	0.18 ± 0.58
*NA_Ruminococcaceae*	−0.16 ± 0.66	−0.18 ± 0.40	−0.54 ± 0.49	−0.98 ± 0.01^NE^	−0.48 ± 0.56
*Prevotella_2*	0.30 ± 0.13 ^a^	0.49 ± 0.52	0.33 ± 0.64	0.59 ± 0.57	−0.09 ± 0.49
*Agathobacter*	0.24 ± 0.13	0.19 ± 0.37	0.59 ± 0.12^a^	0.16 ± 0.66	0.53 ± 0.65
*Lachnospira*	−0.03 ± 0.36	−0.15 ± 0.49	0.04 ± 0.34	−0.95 ± 0.02^NE^	0.38 ± 0.45
*Lachnospiraceae_UCG‐004*	0.09 ± 0.15	0.42 ± 0.44	0.47 ± 0.12^a^	0.14 ± 0.49	0.22 ± 0.66

Abbreviations: ADG, average daily gain; BFT, backfat thickness; DFI, daily feed intake; FCR, feed conversion ratio; RFI, residual feed intake; NE: not estimable.

^a^ Indicate genetic correlations different from zero with a *Z* test (*p* < .05).

With the genera, *r*_g_ ranged from −0.36 ± 0.24 (*Romboutsia*) to 0.32 ± 0.12 (*Streptococcus*) with RFI, from −0.38 ± 0.55 (*Ruminococcaceae_UCG‐005*) to 0.51 ± 0.31 (*Fusicatenibacter*) with FCR, from −0.63 ± 0.45 (*Desulfovibrio*) to 0.60 ± 0.12 (*Faecalibacterium*) with DFI, −0.98 ± NE (*NA_Ruminococcaceae*) to 0.86 ± 0.05 (*Lactobacillus*) with BFT, and from −0.48 ± 0.56 (*NA_Ruminococcaceae*) to 0.73 ± 0.76 (*Lachnospiraceae_UCG‐001*) with ADG. In Table 5, the *r*_g_ of the 22 genera that had at least one significant genetic correlation with the performance traits are presented. The production trait with the highest number of significant *r*_g_ with genera was BFT (11 significant correlations with genera). In addition, three genera had *r*_g_ estimates close to −1 with this trait (*Desulfovibrio*, *NA_Ruminococcacaea*, *Lachnospira*), but *Z*‐tests could not be applied for these cases, as standard errors were not estimable at the borders of the parameter space. The DFI and RFI showed significant *r*_g_ with 7 and 3 genera, respectively, and there were no genera with significant *r*_g_ with ADG and FCR. The genus *Shuttleworthia* had significant genetic correlations with two traits (DFI and BFT), and the genus *Desulfovibrio* had a significant *r*_g_ with RFI and close to −1 with BFT.

From the 10 genera more abundant in the HRFI line, 6 had significant *r*_g_ with at least one production trait, and out of the 42 genera more abundant in the LRFI line, only 7 had significant correlations with the production traits. The other 9 genera with significant genetic correlations with at least one trait were from the 23 genera that had similar abundances between the lines. Distribution between the LRFI and HRFI lines of the abundance of the 22 genera with significant *r*_g_ are presented in Figure 5. The three genera with significant *r*_g_ with RFI (*Streptococcus*, *Desulfovibrio*, and *Prevotella_2*) had significant line abundance differences that were consistent with the sign of the *r*_g_. The genera *Streptococcus* and *Prevotella_2* were more abundant in the HRFI line and had a positive *r*_g_ with RFI, whereas the genus *Desulfovibrio* was more abundant in the LRFI line, and had a negative *r*_g_ with RFI. Out of the 14 genera with significant or very negative genetic correlations with BFT, genera *Blautia*, *Lactobacillus*, and *Dorea* were significantly more abundant in the HRFI line, and had positive *r*_g_ with BFT, and the 5 genera *Prevotella_7*, *Rikenellaceae_RC9_gut_group*, *Desulfovibrio*, *NA_Ruminococcaceae,* and *Lachnospira* were more abundant in the LRFI line, and had negative *r*_g_ with BFT. Of the 7 genera that had significant *r*_g_ with DFI, only the genus *Roseburia* (more abundant in the LRFI line) had significant abundance difference between the two lines, and the sign of the *r*_g_ was not consistent with the line differences.

Box plots showing genera abundances between the LRFI and HRFI lines for the other 53 genera are given in Figure S1.

## 4 DISCUSSION

The objective of the present study was to clarify if some components of pig faecal microbiota have genetic relationships with production and FE traits, taking advantage of data collected in two experimental pig lines divergent for RFI. The approach combined a comparison of the microbiota composition between the genetic lines, and quantitative genetic models to quantify the genetic control on the microbiota components and estimate genetic correlations with traits of interest. These approaches were applied to the subset of genera counts that presented reasonably good properties (number of zeros and Gaussian distribution) to be submitted to linear mixed models. A substantial genetic control for these genera abundances was evidenced with the two approaches, and interesting genetic relationships with the traits of interest were pointed out.

### 4.1 Some genera are under genetic control

Most studies that compared microbiome data of pigs between low and high RFI groups are based on a phenotypic selection of extreme pigs in a population, so most of the reported differences would be driven by phenotypic relationships. In our study differences between animals were established by at least 9 generations of selection, therefore a large proportion of the line differences would result from genetic differences between pigs. Because of the limited size of the lines, the differences can result from an association with the selected trait, or from genetic differences arising by chance (i.e. drift; Hill (1972)). The quantitative models that combine microbiota and production traits for variance components estimations thus provide a complementary approach to evidence genetic relationships between FE and gut microbiota, but its power is more limited than line comparisons.

Some genera differentially abundant between lines pointed out to genera previously reported as associated with feed intake or feed efficiency. Among the most abundant genera that differed between lines, the genus *Lactobacillus* was one of the more abundant ones, with higher abundance associated with high RFI. This genus is well described for its commonness and its important functions in gut health in animals (Dowarah et al., 2017; Valeriano et al., 2017). *Lactobacillus* is the most abundant member of the lactic acid producer bacteria, and is routinely used as a probiotic supplement in the swine nutrition because of its enzymatic activities in the digestion and absorption process of the nutrients in the gut (Kim et al., 2007). Several species of this genus have been reported to have effects on the studied traits (Giang et al., 2011; Shon et al., 2005; Yu et al., 2008). *Lactobacillus* has been reported to be enriched in the faeces of more healthy pigs and positively correlated with feed efficient animals (Bergamaschi, Tiezzi, et al., 2020; Yang et al., 2017). Considering the better health of the LRFI pigs (Chatelet et al., 2018), the lower abundance of *Lactobacillus* in this line was surprising. Conversely, in a study on the faecal microbiota at 80 days of age in Duroc pigs, the genus *Lactobacillus* was reported as one of the four dominant genera in pigs with high RFI from 90 to 160 days of age and not in their low RFI counterparts (Si et al., 2020), which is consistent with the lower abundance of this genus in the LRFI line in our study. Similarly, Verschuren et al. (2018) reported a lower abundance of some OTUs belonging to the *Lactobacillus* genus in low FE than high FE gilts, but the reverse for boars. Overall, the favourable functions of the *Lactobacillus* genus could be partially covered by other genera in the LRFI pigs that showed more diversity than the HRFI animals. *Prevotella*, including *Prevotella_9* and *Prevotella_7,* was the second genus differentially abundant between lines. Si et al. (2020) reported a slightly higher abundance for this genus in animals with low RFI (16.25%) in comparison to animals with high RFI (12.48%), which is in contrast with the higher abundance of the genus *Prevotella_9* in HRFI pigs in our study, but is consistent with the more abundant *Prevotella_7* found in the LRFI line. However, He et al. (2019) also reported a lower abundance of *Prevotella_9* in more feed efficient (15.07%) compared to less feed efficient (17.85%) pigs. The prevalence of members of the *Prevotella* genera is related to their enhancer role in the digestion ability and nutrients extraction from high‐fibre plants (Plummer et al., 2020). This complex and relatively diverse genus seems to contain multiple functions related to the sub‐genera reported in the more recent studies that are not yet clearly identified. The genus *Streptococcus*, more prevalent in the HRFI line, is another member of the lactic acid producer bacteria (du Toit et al., 2014). McCormack et al. (2017) reported a 2‐fold lower abundance of the genus *Clostridium_sensu_stricto_1* in low RFI pigs than high RFI pigs, which is in contrast with our observed higher abundance in the LRFI line.

The results of NMDS confirmed the hypothesis of changes in the intestinal microbial community as a result of selection for feed efficiency. Even though the genera contributions were consistent with their prevalence in the lines (for instance, the genera *Lactobacillus* and *Prevotella_9* had negative loadings on the second axis, which corresponded to the direction of the HRFI line), the extent of the contributions was not related to the abundance in the two lines. For instance, genera from the *Ruminococcaceae* family had an abundance lower than 2% in the LRFI line, but they were among the highest positive contributors to the second axis.

Our results showed significant additive genetic variance for 61% of the analysed genera. Overall, observing significant heritabilities for more than half of the analysed genera, which represented about 97% of the gut microbial communities, suggests that a considerable part of variability of the gut microbial community is under genetic control. However, some heritable genera were shown to differ between lines, but some differentially abundant genera were not heritable, and some heritable genera did not differ between the lines. This last situation could correspond to genera with limited genetic relationship with the selection criterion that would thus not respond to selection and be differentially abundant. The situation of genera that were differentially abundant between lines and not heritable in our study can be related to a limited power of our experimental design to estimate accurately the variance components: only h^2^ estimates higher than 0.125 could be declared significant, so all genera with low heritability would be ignored in our results. Besides, the slight correlation between h^2^ estimates and the average genera abundances found in our study is usually not expected in genetic studies, and is assumed to be due to the dataset truncation (genera with more than 20% of zero were not analysed, which are genera that tend to be the lowest abundant) and consequently missing heritable genera with low abundances. Limited sequencing depth of the microbiota data would cause less precise quantification and high proportion of zeros, that result in imperfect analyses of genera with low abundancies.

Except in few cases, our h^2^ estimates were in the range of previously published values for these genera (Camarinha‐Silva et al., 2017; Chen et al., 2018). For instance, Chen et al. (2018) reported an h^2^ of 0.26 for genus *Turicibacter*, that is higher than our estimate (0.10 ± 0.03). Among the genera that now have sub‐types (*Prevotella*, *Coprococcus* and *Ruminococcus*), we obtained different h^2^ values for the different types. For *Prevotella*, h^2^ ranged from 0.44 ± 0.11 for *Prevotella_1* to 0.01 ± NE (*Prevotella_9*). Chen et al. (2018) have been reported an h^2^ of 0.23 for the genus *Prevotella* and 0.22 for the genus *Coprococcus* that are in agreement with our estimations for the *Prevotella_7* (0.28 ± 0.10) and *Coprococcus_1* (0.32 ± 0.10). The estimated h^2^ for genus *Lactobacillus* (0.24 ± 0.09) was higher than the reported value (0.08) by Chen et al. (2018) and lower than the value (0.34) reported by Camarinha‐Silva et al. (2017). We obtained same h^2^ for the genus *Blautia* (0.39 ± 0.11) as Camarinha‐Silva et al. (2017) (0.33 ± 0.14), and slightly lower h^2^ for the genus *Alloprevotella* (0.22 ± 0.09) than their report (0.34 ± 0.16). Some discrepancies with previously reported estimates could indicate that the genetic determinism of some genera is affected by the study conditions, either animal dependent (breed, age at sampling, etc.) or related to external conditions (feeding, antibiotic distributions, other management choices, etc.), and would need validation in larger and more diverse conditions.

### 4.2 Some genera are genetically correlated with production and FE traits

Obtaining *r*_g_ between genera and performance traits highlights the genetic‐based interaction between feed efficiency components and gut microbiota composition. About 30% of the studied genera had a significant genetic correlation with a studied trait. However, the number of significant *r*_g_ and their magnitudes differed between the five traits. For instance, we could not observe any significant *r*_g_ with FCR and ADG, which might be due to the limited power of the analyses. This indicates that in our study, the strength of the genetic links between genera and ADG or FCR were lower than with the three other traits.

The negative *r*_g_ of the *Streptococcus* genus with RFI and its higher abundance in the HRFI pigs in our study is in agreement with the report of Quan et al. (2018). Similarly, our *r*_g_ estimate with RFI for the genus *Prevotella_7*, and its lower abundance in LRFI pigs, was consistent with the prevalence of the *Prevotellaceae* family in low versus high FCR pigs reported by Quan et al. (2018). Finally, the genus *Desulfovibrio*, that had a negative *r*_g_ with RFI and higher abundance in the LRFI pigs, is known as a sulphate‐reducing bacteria that metabolizes sulphites and sulphates of the diet (Gibson, 1990; Kerr et al., 2011). The genus *Desulfovibrio* was also reported with a negative correlation with feed efficiency traits at the phenotypic level in Large White pigs by Bergamaschi, Tiezzi, et al. (2020). Identifying only three significant *r*_g_ with RFI, and none with FCR, seemed very low numbers given the biological assumptions of the key role of gut microbiota on nutrient availability of the host. However, previous studies also showed limited associations between feed efficiency and single microbiota components (Yang et al., 2017). Besides biological mechanisms, this could be related to maternal genetic and litter effects involved in the variability of the microbial community that could not be fully accounted for in this analysis. When considering DFI, only the genus *Roseburia* showed significant *r*_g_. The positive *r*_g_ with DFI was not in accordance with its higher abundance in the LRFI line, but He et al. (2019) also reported a higher abundance of this *Roseburia* in low FI pigs. Conflict in the line abundances and *r*_g_ also suggests that other factors might be driving this genus abundance at the line level (maternal effects, litter effects), that would deserve further analyses.

The higher number of significant *r*_g_ between genera and BFT could be partly due to the higher h^2^ of BFT, in comparison to the other traits, that could give more power to these estimations. The general composition of backfat in pigs includes water, collagen, and lipids (mainly triacylglycerols) (Wood et al., 1989). Therefore, BFT can be directly affected by the metabolic functions of the microbial composition of the gut. He et al. (2016) have found an association between fatness and OTUs annotated to the genera *Blautia*, *Coprococcus*, and *Ruminococcus* in the caecum samples of pigs. The considerable *r*_g_ of the genera *Blautia*, *Coprococcus_3*, *Coprococcus_1*, and *Ruminococcaceae_UCG_008* with BFT in our result is confirming this results of He et al. (2016). Of the 14 genera with significant *r*_g_ with BFT, 8 genera (*Blautia*, *Coprococcus_3*, *Syntrophococcus*, *Coprococcus_1*, *Marvinbryantia*, *Dorea*, *Shuttleworthia*, and *Lachnospira*) belonged to the *Lachnospiraceae* family. Biddle et al. (2013) argued that *Lachnospiraceae* and *Ruminococcaceae* families have a role of decomposing substrates from indigestible plant materials of the diet (e.g. cellulose and hemicellulose) in the gut. Compounds resulting from such decomposition would be fermented and converted into the acetate, butyrate, and propionate (short‐chain fatty acids—SCFAs) that are absorbable and useable as energy sources by the host (Biddle et al., 2013). The SCFAs also have essential roles in the composition of the gut environment, maintaining electrolyte balance, and providing energy for host cells as well as gut microbiota (Rios‐Covian et al., 2016). Therefore, more availability of SCFAs in the gut environment by the activity of bacteria belonging to the *Lachnospiraceae* and *Ruminococcaceae* families, which have systematic impacts on lipid metabolism and fat storage could justify the chained relationship of these genera with BFT. Given the importance of the BFT as an indicator for carcass payment and reproductive traits of pigs (Roongsitthichai & Tummaruk, 2014), the genetic control of the *Lachnospiraceae* and *Ruminococcaceae* families and the genera belonging to them can have major economic importance in the pig breeding.

### 4.3 α‐diversity indexes are under genetic control and are related to FE traits

Higher microbial diversity is often considered as an attribute of gut health, as animals with the more diverse microbial community are potentially more capable to better deal with pathogenic microbes (Fouhse et al., 2016). It has been more generally linked to increased functional redundancies among the microbial community, which can contribute to a more stable metabolic state and better resilience to face larger variability of feeding resources (Moya & Ferrer, 2016). Therefore, microbial diversity is beneficial for the growth performance and productivity of animals (Fouhse et al., 2016; Hildebrand et al., 2013). This relationship with feed efficiency was confirmed by the negative *r*_g_ between the α‐diversity metrics and the five traits. Negative correlations imply that selecting animals for improved feed efficiency (lower RFI or FCR) will result in increased intestinal microbial community diversity. In the literature, genetic parameters for α‐diversity metrics are rarely reported. Lu et al. (2018), in a study on longitudinal diversity of faecal microbiota in swine, found an h^2^ estimate of 0.04 ± 0.04 for the Shannon index at weaning and 0.18 ± 0.08 at week 15 of age. In another study on rumen microbial features in cattle, an h^2^ of 0.23 ± 0.09 for the Shannon index and 0.19 ± 0.08 for the Simpson index have been reported (Li et al., 2019). Our estimates of h^2^ for both metrics fell into the range of those values. The obtained genetic correlation between the Shannon index and ADG in the present study was lower than −0.53 ± 0.29 reported by Lu et al. (2018). Nevertheless, we have found a stronger *r*_g_ between the Shannon index and BFT than their reports (−0.53 ± 0.23 and −0.45 ± 0.25), but given the standard errors in both studies, our estimates are not statistically different from theirs. Given the genetic properties found in our study and the links reported with gut health and immunity, those synthetic descriptors of gut microbiota composition could be promising traits for selection.

### 4.4 Potential for selection and management in pig production

Our results clearly indicate a genetic basis for part of the gut microbiota composition involved in the variation of feed efficiency (*Streptococcus, Prevotella_7, Desulfovibrio*) and body composition traits (*Lachnospiraceae* family). However, selection to change single microbiota components in order to improve performance traits seems contradictory with the beneficial relationships found between performance traits and microbiota diversity. In that respect, selecting for indicators of microbiota diversity, such as the Shannon index, could be a more generic option. This could also be less dependent on the microbiota specificities due to breeding conditions and sampling characteristics. Indeed, in addition to the genetic, multiple factors can affect the relative abundance of microbiota components and their relationships with traits, including breed and age at sampling (Bergamaschi, Tiezzi, et al., 2020), breeding environment (Le Sciellour et al., 2019), and of course diets (Verschuren et al., 2018). Therefore, more generic indicators of microbiota composition, such as diversity indexes, or mixed models including a microbiability component (Weishaar et al., 2020), might be more relevant for selection. Finally, for some genera (e.g. *Roseburia*) the genetic relationships seemed to be also depend on other factors that could not be accounted for in the present analysis. Deciphering the role of these different factors (genetics, litter and maternal for instance) would clarify the potential for use of these microbiota components to orientate pig performances via different levers of management, including the use of pro‐ and prebiotics, as proposed by Maltecca et al. (2020).

## 5 CONCLUSION

Our results showed substantial effects of genetics on the variability of gut genera community and their relationship with the feed efficiency in pigs. Both analyses of line effect and genetic correlations with production traits revealed a substantial genetic basis for the links between feed efficiency traits and genera and individual diversity of the gut microbial community. The higher diversity in more feed efficient pigs might be related to better gut health and resilience to feed changes. Genera annotated to the *Lachnospiraceae* family had more significant correlations with the studied traits than genera from other families. Functional analyses will be needed to validate the underlying mechanisms. The robustness of these findings requires further validations in different breeding conditions. However, they offer promising perspectives for selection for feed efficiency using gut microbiome composition in pigs.

## CONFLICT OF INTEREST

The authors declare that they have no competing interests.

## Supporting information

Fig S1

Table S1

Table S2
