# Supplementary figures and images for: Genetic relationships between feed efficiency and gut microbiome in pig lines selected for residual feed intake
Source: J Anim Breed Genet. 2021 Feb 26;138(4):491–507. doi: 10.1111/jbg.12539 (PMC8248129; doi:10.1111/jbg.12539)

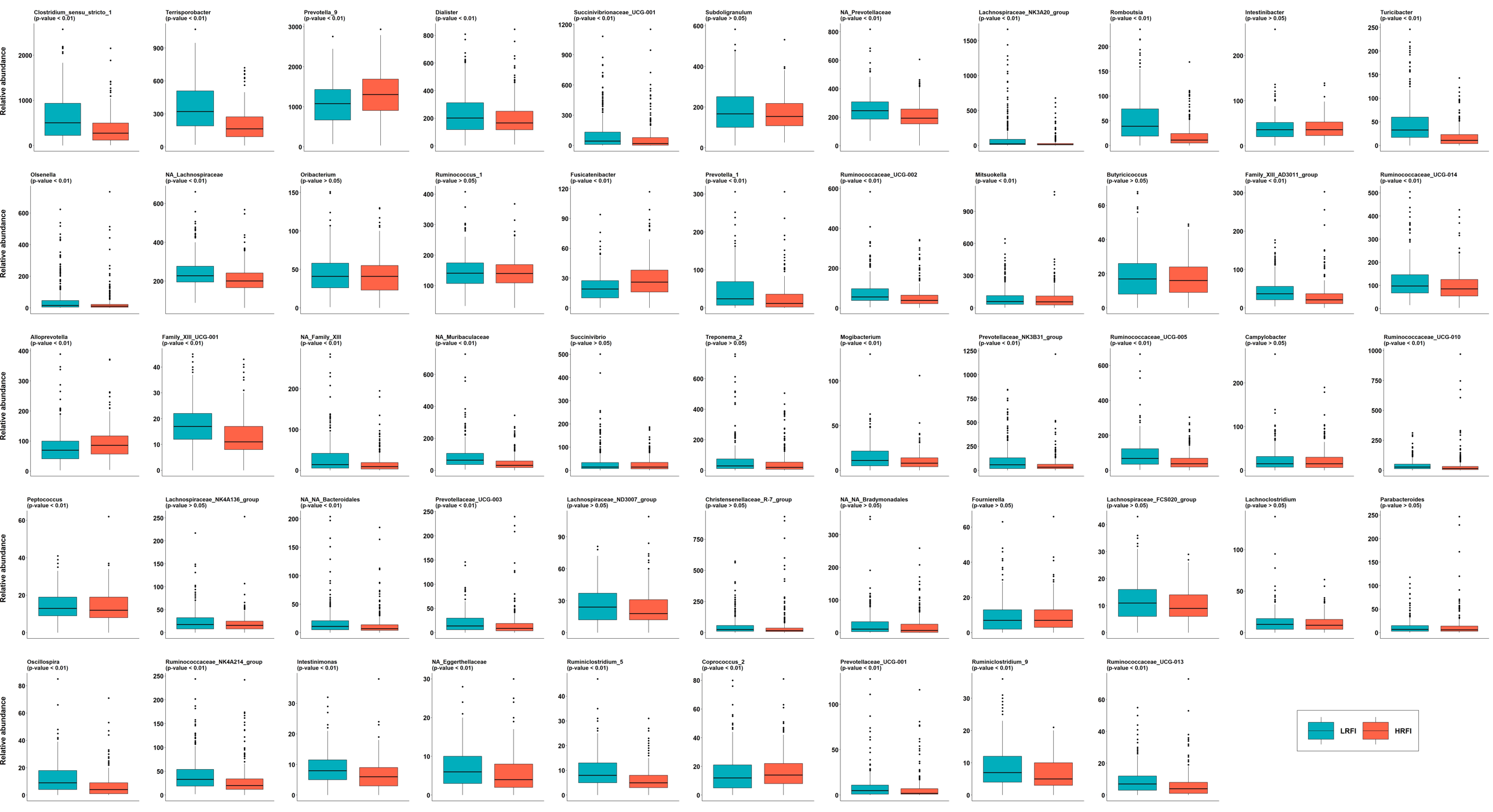

Supplement: Supplementary file 1 — Fig S1 [file JBG-138-491-s001.jpeg]
