# Supplementary material for: Genetic relationships between feed efficiency and gut microbiome in pig lines selected for residual feed intake
Source: J Anim Breed Genet. 2021 Feb 26;138(4):491–507. doi: 10.1111/jbg.12539 (PMC8248129; doi:10.1111/jbg.12539)
Supplement: Supplementary file 2 — Table S1 [file JBG-138-491-s003.docx]

| **Table S1 P-values of the fixed effects**^†^ **tested with linear models on α-diversity indexes and the 75 genera** | | | | | |
| --- | --- | --- | --- | --- | --- |
|  | **BW at Test** | **CG** | **Sex** | **Herd** | **Pen size** |
| **α-diversity index** |  |  |  |  |  |
| Shannon | 0.2109 | 0.0004 | 0.2340 | 0.3416 | <0.0001 |
| Simpson | 0.2197 | 0.0343 | 0.6425 | 0.3446 | 0.0006 |
|  |  |  |  |  |  |
| **Genus** |  |  |  |  |  |
| Clostridium_sensu_stricto_1 | 0.0368 | <0.0001 | 0.8528 | 0.1755 | 0.0056 |
| Prevotella_1 | 0.7835 | <0.0001 | 0.2644 | 0.0579 | 0.0018 |
| Blautia | 0.0341 | <0.0001 | 0.0202 | 0.4815 | 0.0005 |
| Prevotellaceae_NK3B31_group | 0.4067 | <0.0001 | 0.0901 | 0.2988 | 0.012 |
| Lachnospiraceae_NK3A20_group | 0.599 | 0.0004 | 0.3593 | 0.9592 | 0.002 |
| Ruminococcaceae_UCG-008 | 0.001 | <0.0001 | 0.2229 | 0.8803 | 0.6962 |
| Lachnospiraceae_ND3007_group | 0.0217 | 0.0026 | 0.1102 | 0.8725 | 0.2676 |
| Coprococcus_3 | 0.1679 | <0.0001 | 0.166 | 0.8281 | 0.9912 |
| Butyricicoccus | 0.0211 | 0.0002 | 0.0099 | 0.5954 | 0.001 |
| Terrisporobacter | 0.0309 | <0.0001 | 0.6791 | 0.3966 | 0.0001 |
| Syntrophococcus | 0.321 | <0.0001 | 0.5874 | 0.28 | 0.1499 |
| Faecalibacterium | 0.0247 | <0.0001 | 0.0003 | 0.2983 | <0.0001 |
| Coprococcus_1 | 0.6799 | <0.0001 | 0.4349 | 0.8021 | 0.5994 |
| Marvinbryantia | 0.4238 | <0.0001 | 0.086 | 0.3768 | 0.6486 |
| Mitsuokella | 0.2467 | <0.0001 | 0.0811 | 0.0459 | 0.113 |
| NA_Family_XIII | 0.3531 | <0.0001 | 0.6175 | 0.9327 | 0.1163 |
| Prevotella_7 | 0.8175 | <0.0001 | 0.3251 | 0.8211 | 0.3179 |
| Prevotellaceae_UCG-003 | 0.9624 | <0.0001 | 0.2044 | 0.1044 | 0.0659 |
| Romboutsia | 0.4618 | <0.0001 | 0.8058 | 0.0629 | <0.0001 |
| Fusicatenibacter | 0.0106 | 0.0006 | 0.0107 | 0.1828 | 0.0001 |
| Campylobacter | 0.4882 | <0.0001 | 0.0164 | 0.9164 | 0.0258 |
| Olsenella | 0.6392 | <0.0001 | 0.3613 | 0.1878 | 0.2266 |
| Oscillospira | 0.6924 | <0.0001 | 0.022 | 0.232 | 0.0007 |
| Lactobacillus | 0.911 | <0.0001 | 0.6363 | 0.6634 | <0.0001 |
| Roseburia | <0.0001 | <0.0001 | 0.109 | 0.0453 | 0.0312 |
| Succinivibrionaceae_UCG-001 | 0.7886 | <0.0001 | 0.6601 | 0.2291 | 0.0832 |
| NA_Muribaculaceae | 0.3327 | <0.0001 | 0.6308 | 0.3702 | <0.0001 |
| Dorea | 0.0015 | 0.0229 | 0.3121 | 0.7921 | 0.0461 |
| Subdoligranulum | 0.0446 | <0.0001 | 0.0034 | 0.1668 | 0.0021 |
| Alloprevotella | 0.4935 | <0.0001 | 0.606 | 0.4329 | 0.0065 |
| Ruminococcaceae_UCG-014 | 0.2208 | <0.0001 | 0.0059 | 0.0882 | 0.0002 |
| Dialister | 0.7177 | <0.0001 | 0.0477 | 0.0896 | 0.0725 |
| Shuttleworthia | 0.7231 | <0.0001 | 0.4121 | 0.9095 | 0.664 |
| Streptococcus | 0.7774 | <0.0001 | 0.0003 | 0.5731 | 0.0002 |
| NA_Prevotellaceae | 0.6695 | 0.0099 | 0.0223 | 0.26 | 0.0003 |
| Rikenellaceae_RC9_gut_group | 0.5099 | <0.0001 | 0.7212 | 0.4198 | <0.0001 |
| Lachnospiraceae_NK4A136_group | 0.7686 | <0.0001 | 0.0497 | 0.3305 | 0.1102 |
| Desulfovibrio | 0.4229 | <0.0001 | 0.652 | 0.017 | 0.0004 |
| Lachnospiraceae_UCG-001 | 0.0103 | <0.0001 | 0.58 | 0.2758 | 0.9719 |
| Ruminococcus_2 | 0.0342 | <0.0001 | 0.0003 | 0.5176 | 0.0084 |
| NA_Ruminococcaceae | 0.2133 | <0.0001 | 0.6919 | 0.5629 | <0.0001 |
| Treponema_2 | 0.8724 | <0.0001 | 0.1673 | 0.1082 | 0.004 |
| Fournierella | 0.0172 | 0.0027 | 0.19 | 0.3109 | 0.0105 |
| Prevotella_2 | 0.0452 | <0.0001 | 0.1835 | 0.511 | 0.0637 |
| Agathobacter | 0.0014 | <0.0001 | 0.0044 | 0.8655 | 0.0003 |
| Lachnospira | 0.1553 | <0.0001 | 0.0036 | 0.0591 | <0.0001 |
| Ruminococcaceae_UCG-005 | 0.9505 | <0.0001 | 0.3494 | 0.3662 | 0.0001 |
| Lachnospiraceae_UCG-004 | 0.2141 | 0.001 | 0.004 | 0.3512 | 0.0003 |
| Oribacterium | 0.1649 | <0.0001 | <0.0001 | 0.2102 | <0.0001 |
| Ruminiclostridium_5 | 0.2015 | 0.0001 | 0.398 | 0.6998 | <0.0001 |
| Family_XIII_AD3011_group | 0.2655 | <0.0001 | 0.3105 | 0.8536 | 0.0001 |
| Christensenellaceae_R-7_group | 0.2423 | <0.0001 | 0.0837 | 0.9878 | 0.0057 |
| Lachnospiraceae_FCS020_group | 0.425 | <0.0001 | 0.0378 | 0.7094 | 0.2284 |
| NA_NA_Bradymonadales | 0.671 | <0.0001 | 0.4284 | 0.4882 | 0.0083 |
| Family_XIII_UCG-001 | 0.541 | <0.0001 | 0.005 | 0.6487 | 0.2152 |
| Mogibacterium | 0.8338 | <0.0001 | 0.6873 | 0.6786 | 0.0148 |
| Succinivibrio | 0.3089 | 0.0003 | 0.1552 | 0.9261 | 0.2504 |
| Ruminococcaceae_UCG-013 | 0.26 | <0.0001 | 0.8237 | 0.0299 | <0.0001 |
| Intestinimonas | 0.6018 | 0.1622 | 0.9774 | 0.6654 | 0.2347 |
| Turicibacter | 0.2598 | <0.0001 | 0.8787 | 0.0582 | <0.0001 |
| Intestinibacter | 0.0947 | 0.0001 | 0.8015 | 0.9285 | 0.6409 |
| Ruminococcaceae_UCG-002 | 0.0772 | <0.0001 | 0.967 | 0.7606 | <0.0001 |
| NA_Lachnospiraceae | 0.299 | <0.0001 | 0.0009 | 0.2538 | 0.0001 |
| Ruminococcaceae_UCG-010 | 0.7855 | <0.0001 | 0.1223 | 0.3466 | 0.0011 |
| Prevotellaceae_UCG-001 | 0.3501 | <0.0001 | 0.1161 | 0.9331 | 0.0002 |
| Prevotella_9 | 0.0006 | <0.0001 | 0.0142 | 0.9962 | 0.0002 |
| NA_NA_Bacteroidales | 0.4936 | <0.0001 | <0.0001 | 0.8061 | 0.0007 |
| Coprococcus_2 | 0.0083 | <0.0001 | 0.0002 | 0.0376 | <0.0001 |
| Peptococcus | 0.0353 | 0.0048 | 0.161 | 0.3722 | 0.5593 |
| Ruminococcus_1 | 0.7002 | <0.0001 | 0.4908 | 0.5111 | 0.276 |
| NA_Eggerthellaceae | 0.5374 | 0.0007 | 0.1811 | 0.4583 | 0.0245 |
| Ruminiclostridium_9 | 0.8487 | 0.0426 | 0.0031 | 0.6418 | 0.012 |
| Lachnoclostridium | 0.6725 | <0.0001 | 0.1285 | 0.1093 | 0.0614 |
| Ruminococcaceae_NK4A214_group | 0.1475 | <0.0001 | 0.9606 | 0.1203 | <0.0001 |
| Parabacteroides | 0.2206 | <0.0001 | 0.7058 | 0.0308 | 0.3969 |

^†^BW = body weight, CG = contemporary group. Coloured cells show significant effects (P-value < 0.05)
